# Sonographic Characteristics of the Thymus in Healthy Canine Neonates

**DOI:** 10.3390/vetsci13030248

**Published:** 2026-03-06

**Authors:** Emilia Diel, Carsten Staszyk, Kerstin von Pückler, Axel Wehrend

**Affiliations:** 1 Veterinary Clinic for Reproductive Medicine and Neonatology, Justus-Liebig-University, 35392 Giessen, Germany; axel.wehrend@vetmed.uni-giessen.de; 2 Institute of Veterinary-Anatomy, -Histology and -Embryology, Justus-Liebig-University, 35392 Giessen, Germany; carsten.staszyk@vetmed.uni-giessen.de; 3 Veterinary Clinic for Small Animals—Radiology, Justus-Liebig-University, 35392 Giessen, Germany; kerstin.h.pueckler@vetmed.uni-giessen.de

**Keywords:** ultrasonography, thymus, diagnostic imaging, neonatal puppy, mediastinum

## Abstract

The thymus is important for neonatal immune development; however, information on its ultrasonographic appearance in newborn puppies is limited. Because the thymus occupies a substantial portion of the cranial mediastinum in neonates, it may be confused with pathological mediastinal masses during thoracic imaging. Defining its normal ultrasonographic appearance is therefore crucial for accurate clinical interpretation. This study aimed to describe the topographic anatomy and ultrasonographic features of the thymus in neonatal dogs. Ten puppy cadavers were examined. Under ultrasound guidance, the thymus was injected with methylene blue, and its location confirmed by dissection. Subsequently, transcutaneous ultrasonographic examinations were performed in 40 clinically normal puppies aged 1–5 days. In all puppies, the thymus was consistently visualized from the left side of the thorax within the precardiac mediastinum. In the transverse imaging plane, the thoracic portion of the thymus typically appeared triangular, with variations related to respiratory movements and cardiac and vascular pulsations. The thymic parenchyma was predominantly homogeneous and hypoechoic with scattered hyperechoic areas and fine lines. In some cases, the echotexture was more heterogenous, showing fusiform hypo- and hyperechoic linear structures. The thymus was easily differentiated from adjacent thoracic structures due to its distinctive echotexture. The thymus in newborn puppies demonstrates a consistent and recognizable ultrasonographic pattern, allowing reliable identification using transcutaneous ultrasonography. These findings provide a useful reference for clinical and diagnostic evaluation of the neonatal canine thymus.

## Simple Summary

The thymus is an important organ for the immune system in newborn puppies, but its appearance on ultrasound is not well described. In this study, we examined the thymus in healthy neonatal dogs using transcutaneous thoracic ultrasonography. The anatomical position of the thymus was first confirmed in cadavers and then evaluated in live puppies during the first five days of life. In all animals, the thymus could be clearly identified and showed a consistent location and characteristic ultrasound appearance. These results provide a practical reference for veterinarians and may help distinguish normal thymic findings from pathological changes in neonatal dogs.

## 1. Introduction

As a vital component of the immune system, the thymus, a primary lymphoid organ, begins its development in dogs during the fetal stage, around day 27 of gestation. Dogs are generally considered to possess a functionally active immune system at or near birth, although maturation of adaptive immunity continues postnatally [1].

In the dog, the thymus reaches its maximum absolute size around four to six months of age, after which it undergoes a physiological, age-dependent involution. This process is characterized by a gradual reduction in mass and structure, with replacement by adipose tissue [1,2]. The bilobed thymus, with the left lobe significantly larger than the right, is situated in the cranial thoracic cavity between the cranial lung lobes, extending from the cranial margin of the pericardium to the thoracic inlet, with a small portion protruding ventrally to the trachea [3]. Macroscopically, the thymus appears as a lobulated structure with a pink to grey, glassy appearance [3,4].

Although conventional radiography, CT, and MRI are considered the primary imaging modalities for imaging the chest of children in human pediatrics, ultrasonography can provide useful and, in some cases, superior information with the advantage of avoiding exposure to radiation [5]. In pregnant women, assessment of the fetal thymus by ultrasonography during the different stages of pregnancy has become common practice. The thymus is best visualized in the three-vessel and trachea view, where identification of the internal mammary arteries, serving as the lateral borders of the “thy-box”, allows reliable identification and measurement of the thymus [6]. It has been shown that a small fetal thymus on ultrasound may be associated with different disorders during pregnancy like preterm birth and neonatal sepsis [7].

Given the anatomical and developmental similarities of the thymus across mammalian species, insights from pediatric imaging may provide valuable conceptual guidance for veterinary applications. Within veterinary medicine, initial studies have described the ultrasonographic evaluation of the thymus in puppies in the postnatal phase [8], and ultrasonographic examination of the fetal thymus during gestation have also been reported [9,10]. It has been demonstrated that the thymus in puppies responds to disease with a reduction in size. Following inoculation with the canine distemper virus, puppies exhibited a five- to sixfold reduction in thymus size within ten days, as determined by necropsy [11].

To reliably differentiate between physiological and pathological processes, a thorough understanding of the sonomorphology of the thymus in healthy puppies is essential. The aim of this study was to characterize the normal sonomorphological features of the thymus in healthy neonatal puppies during the first days of life. These findings may serve as a foundation for future studies establishing quantitative reference values.

## 2. Materials and Methods

All procedures were performed in accordance with national and institutional guidelines. Ethical approval was not required, as the study involved either routine clinical examinations or cadavers from animals that died or were euthanized for reasons unrelated to the study. Written informed consent was obtained from all owners prior to inclusion.

The study was conducted in two phases: (1) a cadaveric validation phase and (2) a clinical ultrasonographic evaluation phase. In the first phase of the study, ten puppies (aged one to five days) that had either died or been humanely euthanized for unrelated clinical reasons were utilized. Cadavers were examined within 12 h post-mortem and were stored either at room temperature or under refrigeration, but were not frozen prior to evaluation. Their bodies were assessed according to the same examination protocol used in phase 2, including standardized ultrasound position, settings, and imaging procedures detailed below. Once the thymus had been identified via ultrasonography, methylene blue (Merck KGaA, Darmstadt, Germany) was introduced into the tissue under real-time imaging. The cadavers were then anatomically explored to validate the ultrasound findings.

The second phase involved sonographic evaluation of 40 live newborn puppies between one and five days old. Among the group, 23 were female and 17 were male. The cohort comprised various breeds, also including mongrel puppies, with a predominance of mesocephalic individuals, while brachycephalic puppies were also represented. Puppies were delivered either via monitored spontaneous parturition or by cesarean section. Only clinically healthy neonates were included, defined as the absence of congenital anomalies, normal mucous membrane coloration, intact neurologic reflexes, appropriate muscle tone, and no visible evidence of respiratory or cardiovascular compromise. The examinations were performed during routine neonatal health checks focusing on pulmonary assessment. Ultrasound data were obtained during the animals’ first clinical visit or immediately following birth.

All ultrasound examinations were conducted by a single examiner. Thoracic sonography was conducted in B-mode. The puppies were examined using a stationary ultrasound system (Canon Aplio a (CUS-AA000) Canon Medical Systems Europe B.V., Zoetermeer, The Netherlands) equipped with a linear 4.5–17 MHz transducer (17LH7).

Examinations were performed with the puppies in right lateral recumbency. The skin over the left side of the thorax was moistened with isopropanol and supplemented with ultrasound gel. Imaging parameters, including gain and focal depth, were individually adjusted during each examination to optimize image quality for each puppy.

For imaging the thymus, a transverse and longitudinal imaging plane were selected. To visualize the cross-section of the thymus, the left forelimb was gently pulled cranially, and the ultrasound probe was positioned perpendicular to the skin surface and parallel to the longitudinal axis of the ribs in the first to fourth intercostal space. By inclining the probe caudally, the cranial margin of the pericardium became visible and served as an orientation point. Additional landmarks included the brachiocephalic trunk, the left subclavian artery and the cranial vena cava, which are located dorsal and slightly medial to the thymus in the cranial part of the mediastinum.

For the longitudinal assessment, a left parasternal acoustic window through the costal cartilage was selected to avoid acoustic shadowing caused by the ribs.

## 3. Results

**Figure 1:**
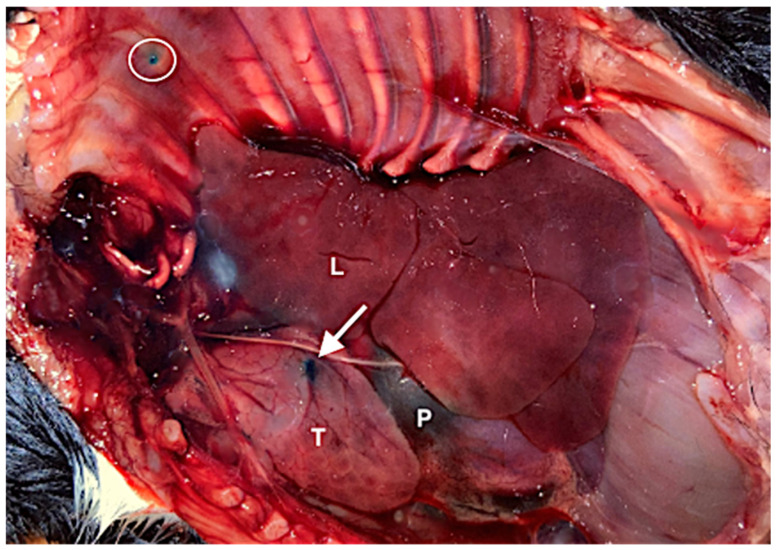
Representative image from the cadaver study. The thorax of a Great Dane puppy that died during dystocia was opened on the left side. Visible structures include the left lung (L), pericardium (P), and the thymus (T), which is partially stained with methylene blue (arrow). The white circle indicates the puncture site in the thoracic wall.

In all animals examined, the thymus was clearly identifiable during anatomical dissection (Figure 1). Ultrasonographically, the thymus was consistently visualized from the left side of the thorax within the cranial mediastinum in all puppies. In transverse view the thymus typically appeared triangular, although variations in its contour were observed related to respiration and cardiac and vascular pulsation.

**Figure 2:**
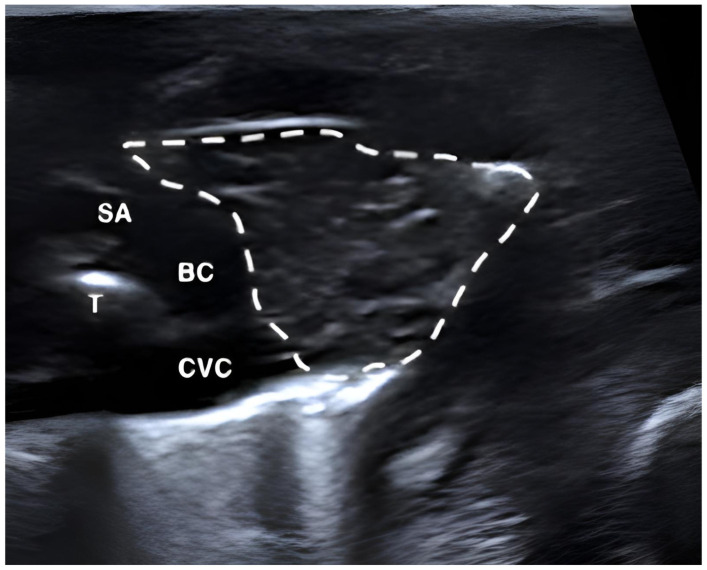
Ultrasonographic image of the thymus (outlined) in a live, healthy mongrel puppy delivered by cesarean section. Transcutaneous ultrasonography was performed from the left side of the thorax in transverse orientation, with the image acquired at the cranial aspect of the mediastinum. BC = brachiocephalic trunk, CVC = cranial vena cava, SA = left subclavian artery, T = trachea.

**Figure 3:**
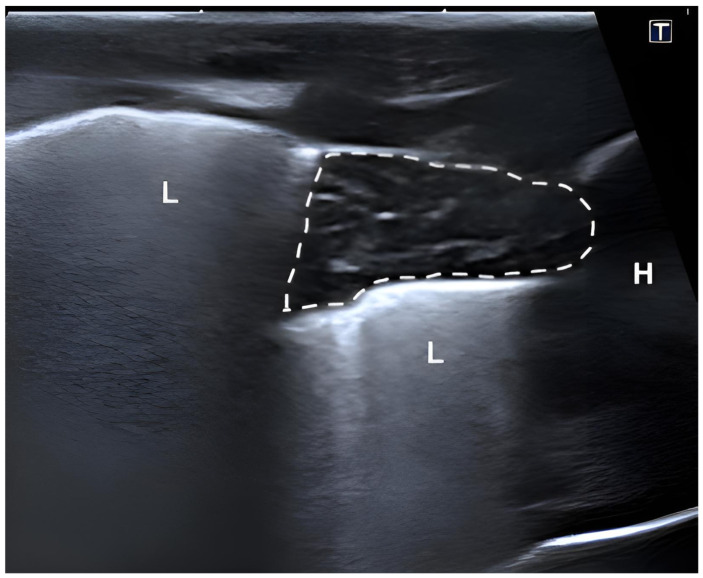
Transcutaneous ultrasonographic image of the thymus (outlined) in a live, healthy Golden Retriever puppy delivered via cesarean section. Imaging was performed from the left thoracic wall in transverse orientation, cranial to the heart. The thymus appears as a hypoechoic, lobulated structure with fine internal echogenic septa. L = lung, H = heart.

The thymic parenchyma predominantly exhibited a homogeneous hypoechoic echotexture with interspersed hyperechoic areas and fine linear structures, creating a reticular or striated pattern. Some puppies showed a more heterogeneous appearance with spindle-shaped hypo- and hyperechoic linear structures distributed throughout the parenchyma. The thymus was readily distinguishable from adjacent thoracic structures due to its characteristic echotexture. In cross-sectional view, the thymus was located between the sternum and the major thoracic vessels, including the cranial vena cava, brachiocephalic trunk, and left subclavian artery in the cranial mediastinum (Figure 2). In more caudally located transverse sections, the thymus appeared triangular and was surrounded by pulmonary tissue (Figure 3).

**Figure 4:**
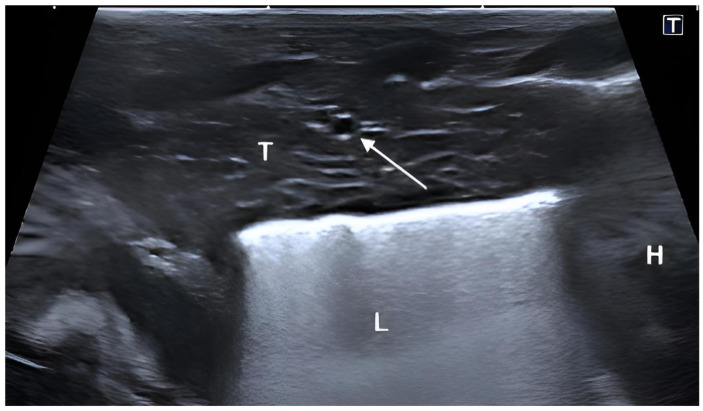
Transcutaneous ultrasonographic image of the thymus (T) in a live, healthy Golden Retriever puppy delivered via cesarean section. The image shows the thymus view from the parasternal window with a longitudinal imaging plane with the image acquired at the cranial aspect of the mediastinum. The thymus appears as an elongated, hypoechoic structure with fine echogenic septa. The arrow indicates an intrathymic blood vessel appearing as a hyperechoic structure. L = lung, H = heart.

Longitudinal visualization was also achieved using a parasternal probe position. In this view, the thymus appeared elongated and demonstrated a predominantly homogeneous echotexture with scattered fine hyperechoic lines throughout the parenchyma. Additionally, this view clearly showed that the thymus lies directly cranial to the heart, confirming its close anatomical relationship with the cranial cardiac border (Figure 4).

## 4. Discussion

This study provides a detailed description of the topographic anatomy and sonographic appearance of the thymus in neonatal puppies. In all animals examined, the thymus was clearly identifiable both sonographically and anatomically, confirming that transcutaneous thoracic ultrasonography is a reliable method for identifying this organ during the first days of life.

The homogenous echotexture observed in the neonatal canine thymus closely resembles findings reported in human neonates. In infants, the thymus typically appears hypoechoic with fine echogenic septa representing connective tissue and intrathymic vessels, which create a characteristic echo pattern facilitating differentiation from surrounding mediastinal structures [12,13].

Age-related changes in human thymic echotexture have been documented. In infants under 2–3 years, the thymus is predominantly hypoechoic with thin septa, in children aged 2–14 years, echogenic foci and a “starry sky” appearance become increasingly prominent and in older children and adults, the thymus becomes largely hyperechoic due to fatty infiltration [14]. Since the present study did not include animals of different age groups, no conclusions can be drawn regarding age-related changes in thymic sonomorphology in puppies.

Although radiography, CT, and MRI remain primary modalities for evaluating the pediatric thorax, ultrasonography has become an important complementary tool, offering valuable information without exposure to ionizing radiation [15]. In pregnant women, the fetal thymus is routinely assessed, most reliably visualized in the three-vessel and trachea view using the internal mammary arteries as lateral borders of the “thy-box” [16]. In postnatal imaging, optimal thymic visualization relies on careful transducer selection and precise probe positioning to find the best acoustic windows. It has been further noted that the relatively unossified thorax and large thymus in neonates and infants facilitate imaging of the anterior chest and mediastinum. Our observations closely align with these considerations, confirming the importance of both technical approach and neonatal thoracic anatomy for high-quality thymic imaging [5].

Hight-definition ultrasonography was used to assess embryonic and fetal development in twelve brachycephalic bitches and visualization of the fetal thymus was reported from approximately day 40 of gestation. Changes in echogenicity were observed as gestation progressed [9]. By contrast, the fetal thymus was identified from day 48 of gestation onward using conventional ultrasonography, and no alterations in morphological characteristics or echogenicity were observed until parturition [10]. These differences may be attributable to variations in imaging resolution and ultrasound technique rather than true biological variation.

The ultrasonographic evaluation of the thymus in neonatal puppies was also investigated, with imaging performed through the cranial intercostal spaces, the latter being identified as the optimal plane for transverse imaging of the thymus. Our qualitative observations regarding the neonatal thymus are largely consistent with these findings. Both studies confirm that the thymus is readily identifiable sonographically in puppies and report a homogeneous parenchymal echotexture [8].

Similar to humans, the thymus in puppies responds to disease with marked reduction in size. For example, following inoculation with canine distemper virus, thymic volume decreased five- to sixfold within ten days, as confirmed by necropsy [11]. Establishing normative sonomorphological baselines, as provided in the present study, is therefore crucial for identifying pathological changes and for assessing neonatal immune status, as well as for differentiating the thymus from mediastinal masses, evaluating potential immune compromise, and avoiding misinterpretation of normal thymic tissue as thoracic pathology [17].

Despite many advantages, ultrasonographic evaluation of the thymus has several limitations. The presence of air surrounding the thorax can interfere with sound wave transmission and reduce image quality [14]. In canine fetal imaging, assessment is further challenged by small patient size, particularly in puppies of small breeds, as well as by pronounced movement, which can hinder stable probe positioning. Moarabi et al. (2021) implemented a sedation protocol in their study, which can overcome these movement-related difficulties, although this approach is considerably more invasive [8].

Furthermore, while ultrasonography is widely used for thymic size estimation in children, sonographic measurements have been shown to deviate from volumetric data obtained at autopsy, highlighting inherent limitations in the accuracy of ultrasound-based size assessment [15].

Also, visualization of the fetal thymus was significantly reduced in bitches with a body condition score (BCS) greater than 4, likely due to increased adipose tissue thickness and reduced image resolution [10]. For visualization of the fetal thymus and reproducibility of measurements of thymus size MRI appears superior to ultrasound when available [16]. For evaluation of the thymus in young and adult dogs, computed tomography has been described in veterinary medicine [2].

This study focused on a qualitative assessment of the thymus during the first days of life. No systematic quantitative measurements were obtained, representing an important limitation that prevents the establishment of reference values for thymic size and morphology. Breed-specific differences were not evaluated, and respiratory and patient motion occasionally limited assessment of thymic shape, introducing additional variability. Despite these constraints, the study provides a detailed description of the thymus’ ultrasonographic appearance, which may serve as a foundation for future research. Future studies should aim to quantify thymic dimensions, investigate breed-related variations, and employ multimodal imaging approaches, which are particularly important for the early detection of thymic abnormalities and systemic disorders [17].

## 5. Conclusions

This study confirms that the thymus in neonatal puppies can be reliably identified using transcutaneous thoracic ultrasonography. The organ consistently exhibits a predominantly homogeneous hypoechoic parenchyma with fine hyperechoic septa, allowing clear distinction from surrounding thoracic structures and tissue. These findings may facilitate early clinical decision-making in neonatal patients.

## Data Availability

The original contributions presented in this study are included in the article. Further inquiries can be directed to the corresponding author.

## Abbreviations

The following abbreviations are used in this manuscript:

BCS	Body Condition Score
B-mode	Brightness Mode
